# Identification of predictive factors interacting with heart rate reduction for potential beneficial clinical outcomes in chronic heart failure: A systematic literature review and *meta*-analysis

**DOI:** 10.1016/j.ijcha.2022.101141

**Published:** 2022-10-29

**Authors:** Akira Yamashina, Masanori Nishikori, Hiroaki Fujimito, Koji Oba

**Affiliations:** a Department of Cardiology, Tokyo Medical University, Tokyo, Japan; b Department of Health Sciences, Kiryu University, Gunma, Japan; c Medical Affairs Division, Ono Pharmaceutical Co., Ltd., Osaka, Japan; d Interfaculty Initiative in Information Studies, The University of Tokyo, Tokyo, Japan; e Department of Biostatistics, School of Public Health, Graduate School of Medicine, The University of Tokyo, Tokyo, Japan

**Keywords:** Bayesian analysis, Chronic heart failure, Heart rate, Meta-analysis, Heart failure with reduced ejection fraction, Predictive factors, AF, atrial fibrillation, CrI, credible interval, CV, cardiovascular, HF, heart failure, HR, heart rate, HFrEF, HF with reduced ejection fraction, LVEF, left ventricular ejection fraction, MI, myocardial infarction, NYHA, New York Heart Association, PRISMA, Preferred Reporting Items for Systematic Reviews and Meta-Analyses, RR, risk ratio, T2DM, type 2 diabetes mellitus, WHF, worsening heart failure

## Abstract

**Background:**

There is an absence of clinical evidence on what factors modify the effect of heart rate (HR)-reducing treatment on mortality and morbidity in symptomatic heart failure patients with reduced ejection fraction (HFrEF). We performed a Bayesian *meta*-analysis and *meta*-regression to identify predictive factors that interact with HR-reducing therapy.

**Methods:**

A systematic review was performed to identify randomized placebo-controlled trials that enrolled symptomatic HFrEF patients. The primary objective was to evaluate how different predictive factors modify the efficacy of HR-reducing therapy on clinical outcomes. Secondary objectives included the evaluation of subgroups stratified by a HR reduction threshold of 10 bpm.

**Results:**

Data from 20 studies were synthesized and HR-reducing therapy was responsible for 16.7 %, 16.4 %, and 21.1 % risk reductions in all-cause mortality, cardiovascular (CV)-related mortality, and rehospitalization due to worsening HF (WHF), respectively. Empirical Bayes *meta*-regression showed that type 2 diabetes mellitus (T2DM) significantly modified the efficacy of HR-reducing therapy on all-cause mortality (slope = 0.012 in log risk ratio (RR) per 1 %-unit [95 % credible interval (CrI) 0.004, 0.021]) and CV-related mortality (0.01 in log RR per 1 %-unit [95 % CrI 0.0003, 0.0200]). There were insufficient studies to perform a *meta*-regression when stratifying by a HR reduction threshold of 10 bpm; however, when including all studies, we observed a significant effect modification for rehospitalization due to WHF (p = 0.004).

**Conclusions:**

This *meta*-analysis focused on the central tenet of HR-reducing therapy and revealed that T2DM is a predictor of HR-reducing treatment effect on all-cause mortality and CV-related mortality in HFrEF patients.

## Highlights

• We used Bayesian statistics to illustrate factors that affect HR reduction therapy.
• HR-reducing therapy was associated with significant reductions in risk.
• Presence of comorbid T2DM significantly affected mortality risk.

## 1 Introduction

Chronic heart failure (HF) is a life-threatening clinical syndrome characterized not only by cardiac dysfunction but also by various other systemic disorders and comorbidities [1], [2], [3]. In 2021, the burden of HF across 195 countries was reported with data showing that the global number of cases increased by 91.9 % from 33.5 million in 1990 to 64.3 million in 2017 [4]. However, the international prospective REPORT-HF registry suggests there are regional differences in treatment and medical management [5]. Hospitalization for and management of acute HF must take into consideration the etiology and precipitants of HF, various relevant patient- and disease-related characteristics, and the presence of comorbidities. Although the objective of therapy is primarily to treat the symptoms of HF that are affecting a patient’s functional capacity and quality of life, therapy should also improve a patient’s clinical status by suppressing pathological cardiac remodeling (i.e., neurohormonal activation) to improve prognosis [6].

HF leads to increased cardiac contractility and heart rate (HR) by decreasing the carotid baroreceptor response and subsequently increasing sympathetic nervous activity [7]. Many studies have shown an association between HR and clinical outcomes in patients with HF. In patients with chronic HF, an increase in HR of 10 beats per minute (bpm) was associated with an 8 % increase in the risk of cardiovascular death or HF hospitalization regardless of their ejection fraction [8], [9]. In addition, a previous *meta*-analysis reported a significant association between the magnitude of HR reduction (a decrease in HR of 5 bpm) and survival benefit of beta-blockers (18 % reduction in risk of death) in patients with HF [10]. Another more recent *meta*-analysis study reported a reduction in mortality in patients that have HF with reduced ejection fraction (HFrEF) in sinus rhythm who were treated with beta-blockers, regardless of their pre-treatment HR, and that achieving a lower HR was associated with a better prognosis [11].

Therefore, given HR is a factor that affects prognosis, it is more clinically meaningful to identify which patient- and disease-related factors interact with HR-reducing treatment to modify the clinical outcomes in patients with HFrEF who are receiving standard HF therapy. In the SHIFT trial it was suggested that baseline factors (i.e., baseline HR, background therapy, cardiac parameters, and medical history) may interact with HR-reducing treatment [12]. However, the relationship between a patient’s background characteristics and the effect of HR reduction on prognosis has yet to be sufficiently evaluated. It was hypothesized that several patient and disease-related factors including demographic characteristics, comorbidities, and biomarker levels may interact with therapy-induced HR reduction to predict clinical outcome in patients with chronic HFrEF

The overall objective of this systematic literature review and *meta*-analysis is to investigate the interaction between predictive factors (e.g., age, sex, comorbidities, causes and complications of HF, concomitant treatment, and other baseline factors) with HR-reducing treatment and how it influences the clinical outcome in HFrEF patients. The primary study objective is to evaluate how different predictive factors modify the efficacy of HR-reducing treatment, regardless of a drug’s mechanism of action, in HFrEF patients. The secondary study objective includes evaluating how different predictive factors modify the efficacy of HR-reducing treatment in subgroups stratified by a HR reduction threshold of 10 bpm.

## 2 Methods

### 2.1 Protocol and registration

This study was registered at UMIN under the identifier number (UMIN000043495; https://upload.umin.ac.jp/cgi-open-bin/ctr/ctr_view.cgi?recptno=R000049651).

### 2.2 Search strategy

The methodology and results of this study are reported according to the Preferred Reporting Items for Systematic Reviews and Meta-Analyses (PRISMA) (**Text A.1**) [13]. Two independent reviewers (from Edanz) conducted the search, screened all studies for eligibility, performed data extraction, and assessed the risk of bias for each included study. All disagreements were resolved by consensus or by consultation with a third reviewer if necessary. PubMed, EMBASE, and Cochrane CENTRAL databases were searched, and the search string used in this systematic review was as follows: (chronic heart failure) AND (heart rate); AND (left ventricular contractile dysfunction) OR (reduced ejection fraction) OR (heart failure with reduced ejection fraction); NOT (acute) OR (acute decompensated) OR (acute decompensated heart failure) OR (preserved) OR (heart failure with preserved ejection fraction).

### 2.3 Study eligibility

The inclusion criteria were as follows: studies that were randomized and placebo-controlled clinical trials; studies involving symptomatic HFrEF patients aged ≥ 18 years; studies investigating the effect of HF therapies on change in HR and/or other clinical outcomes; studies published in English and with full text available; and studies published between database inception and December 2020. Studies that fulfilled the following criteria were excluded from the literature review: case-control studies, observational studies, studies not investigating HR-reducing therapy, studies with no quantitative data or measurable outcomes, studies with incomplete or qualitative data alone, and reviews and collections of conference abstracts.

### 2.4 Quality assessment

The Cochrane Risk of Bias 2.0 tool was used to assess the methodological quality of the studies to be included [14].

### 2.5 Data extraction

The following data, if available, were extracted: study ID, study title, year of publication, study design, sample size, study duration, patient demographic characteristics (age, sex, body mass index, race, and New York Heart Association [NYHA] class), prior HF therapy, baseline characteristics (HR, left ventricular ejection fraction [LVEF], blood pressure, brain natriuretic peptide, serum creatinine, and estimated glomerular filtration rate), HF characteristics (atrial fibrillation [AF], ischemia, prior myocardial infarction [MI], hypertension-induced, valvular disease, coronary artery disease, and dilated cardiomyopathy), comorbid disorders (type 2 diabetes mellitus [T2DM], hypertension, chronic obstructive pulmonary disease, and prior stroke), and outcome measures (all-cause mortality, cardiovascular [CV]-related mortality, hospitalization due to worsening HF [WHF], incidence of major adverse cardiovascular events [classically defined as a composite of nonfatal stroke, nonfatal MI, and CV death], and any composite outcomes). Post-hoc and secondary analyses of the studies identified in our literature search were also searched for any missing data.

### 2.6 Statistical methods

The data were synthesized using an empirical Bayesian random effect *meta*-analysis to estimate the overall effect on a clinical outcome (all-cause mortality, CV-related mortality, and rehospitalization due to WHF). A restricted maximum likelihood using a Bayesian framework was used for estimating heterogeneity and posterior distributions in this *meta*-analysis as it allowed for the approximation of posterior distributions using priors derived from the data. All eligible studies were combined to estimate the risk ratio (RR), log(RR), and 95 % credible interval (CrI) on each of the outcomes studied. Heterogeneity between studies was assessed using the *I^2^* metric (with the alpha level set at 0.05). Subgroup analysis was conducted based on studies in which HR was reduced by ≥ 10 bpm and those in which HR was reduced by < 10 bpm [8]. For this subgroup analysis, we used the same restricted maximum likelihood principle as in the main analysis.

An empirical Bayes random effects *meta*-regression was used to evaluate the predictive factors of HR-reducing therapies on clinical outcomes using a maximum marginal likelihood method. Here, a *meta*-regression was conducted for all eligible studies where at least 80 % of the predictive factor data were available in 10 or more studies [15]. Prior distributions were calculated from the data and included to model posterior estimates using an empirical Bayes estimator (without any predefined priors) and inputting priors that were derived from the data.

The strength and direction of various associations between the various risk factors and clinical outcomes were described as follows: log(RR) for *meta*-regression models represents a change in log(RR) when a predictor increases by a 1 %-unit rather than a categorical presence or absence. The only exception was LVEF, which is a 1 % increase in LVEF; a positive log(RR) implies that increasing the percent of that factor will increase the risk of an outcome (i.e., it reduces the risk-lowering effect of HR-reducing therapy), whereas a negative log(RR) implies that increasing the percent of that factor will decrease the risk of an outcome (i.e., it increases the risk-lowering effect of HR-reducing therapy). Bayesian posterior probability p-values were estimated using the maximum probability of effect method [16]. *P* values < 0.05 were considered statistically significant. When constructing *meta*-regression plots, a *P* value of < 0.1 was considered a factor of interest for evaluating the robustness of the slope profile.

Sensitivity analyses were performed to check the primary pooled analysis. In addition, sensitivity analyses were conducted with subjective priors to determine the impact of individual studies on the pooled result. A leave-one-out cross-validation was performed to cross-validate the results and further assess the sensitivity of the *meta*-analysis and the risk of bias in individual studies. Funnel plots and the Egger’s test were used to assess publication bias in conjunction with the leave-one-out cross-validation data. The alpha level for plot asymmetry was set at 0.05.

R version 4.2 (R Foundation for Statistical Computing, Vienna, Austria) and RevMan 5.3 (The Cochrane Collaboration, London, UK) were used for statistical analysis. Forest plots were made in the *metafor* package in R [17]. Bayesian posterior probability *p* values were estimated using the *bayestestR* package in R [16].

## 3 Results

### 3.1 Data extraction

A total of 2,799 records were identified through database searching. After removing duplicates, 2,245 article titles and abstracts were reviewed, with a further 1741 articles excluded, after which 60 full-text articles were reviewed, and 20 articles were included in the *meta*-analysis [12], [18], [19], [20], [21], [22], [23], [24], [25], [26], [27], [28], [29], [30], [31], [32], [33], [34], [35], [36]. The PRISMA flow diagram is shown in **Fig. A.1** and all studies included in the primary *meta*-analyses (n = 20) were randomized controlled trials that used a comparative trial design of intervention (HR-reducing therapy) versus placebo. The overall characteristics of the studies included in this *meta*-analysis are listed in Table 1. Furthermore, we searched all available post-hoc and secondary analysis publications for missing data (**Table A.1**).

**Table 1:** Studies included.

**Title**	**Trial Name**	**First Author**	**Year**	**Citation**	**Main Inclusion Criteria**	**Treatment**	**Number of patients**
A trial of the beta-blocker bucindolol in patients with advanced chronic heart failure	BEST	BEST study group	2001	N Engl J Med. 2001; 344(22): 1659–67.	NYHA class III–IV, LVEF% ≤0.35, HR > 50 beats/min, SBP > 80 mmHg	Bucindolol (target 50 mg/day) versus placebo	Treatment: 1354; Placebo: 1354
Dose-response of chronic beta-blocker treatment in heart failure from either idiopathic dilated or ischemic cardiomyopathy. Bucindolol Investigators	–	Bristow	1994	Circulation. 1994;89 (4): 1632–42	NYHA class I-IV, LVEF% ≤0.40	Bucindolol 12.5, 50, or 200 mg/day versus placebo	Treatment: 38/32/35; Placebo: 34
Effect of carvedilol on outcome after myocardial infarction in patients with left-ventricular dysfunction: the CAPRICORN randomized trial	CAPRICORN	CAPRICORN study group	2001	Lancet. 2001;357 (9266): 1385–90.	LVEF% ≤0.40, HR > 60 beats/min, SBP > 90 mmHg	Carvedilol (6.25 mg/day) versus placebo	Treatment: 975; Placebo: 984
Treatment of heart failure with celiprolol, a cardioselective beta blocker with beta-2 agonist vasodilatory properties	CELICARD	Witchitz	2000	Am J Cardiol. 2000; 85(12): 1467–71.	Age > 18 y, NYHA class II-IV, LVEF% ≤0.40, HR > 55 beats/min, SBP > 100 mmHg	Celiprolol (target: 100 mg/day) versus placebo	Treatment: 62; Placebo: 62
A randomized trial of /-blockade in heart failure the Cardiac Insufficiency Bisoprolol Study (CIBIS)	CIBIS-I	CIBIS study group	1994	Circulation. 1994;90(4): 1765–73.	NYHA class III-IV, LVEF% ≤0.40, HR > 65 beats/min, SBP < 160 mmHg	Bisoprolol (target: 10 mg/day) versus placebo	Treatment: 320; Placebo: 321
The Cardiac Insufficiency Bisoprolol Study II (CIBIS-II): A randomized trial	CIBIS-II	CIBIS study group	1999	Lancet. 1999;353 (9146): 9–13	NYHA class III–IV, LVEF% ≤0.35, HR > 60 beats/min, SBP > 100 mmHg	Bisoprolol (target: 10 mg/day) versus placebo	Treatment: 1327; Placebo: 1320
Effect of carvedilol on survival in severe chronic heart failure	COPERNICUS	Packer	2001	N Engl J Med. 2001; 344(22): 1651–8.	NYHA class III–IV, LVEF% ≤0.25, HR > 68 beats/min, SBP > 85 mmHg	Carvedilol (target dose of 25 mg BID) versus placebo	Treatment: 1156; Placebo: 1133
Effects of nebivolol on left ventricular function in elderly patients with chronic heart failure: results of the ENECA study	ENECA	Edes	2005	Eur J Heart Fail. 2005;7(4): 631–9.	Age ≥ 65 y, NYHA class II–IV, LVEF% ≤0.35, HR > 50 beats/min	Nebivolol (10 mg/day) versus placebo	Treatment: 134; Placebo: 126
Beneficial effects of metoprolol in heart failure associated with coronary artery disease: A randomized trial	–	Fisher	1994	J Am Coll Cardiol. 1994; 23(4): 943–50.	NYHA class II-IV, LVEF% ≤0.40, HR > 60 beats/min	Metoprolol (target: 50 mg BID) versus placebo	Treatment: 25; Placebo: 25
Long-term beta-blocker vasodilator therapy improves cardiac function in idiopathic dilated cardiomyopathy: a double-blind, randomized study of bucindolol versus placebo	–	Gilbert	1990	Am J Med. 1990; 88(3): 223–9.	Age 18–80 y, LVEF% ≤0.40, SBP > 80 mmHg	Bucindolol (target: 100 mg BID) versus placebo	Treatment: 14; Placebo: 9
Efficacy and safety of ivabradine in Japanese patients with chronic heart failure	J-SHIFT	Tsutsui	2019	Circ J. 2019;83(10): 2049–60.	Age > 20 y, NYHA class II-IV, LVEF% ≤0.35, HR ≥ 70 beats/min	Ivabradine (target: 2.5–7.5 mg BID) versus placebo	Treatment: 127; Placebo: 127
Double-blind, placebo-controlled study of the long-term efficacy of carvedilol in patients with severe chronic heart failure	–	Krum	1995	Circulation. 1995;92(6): 1499–506.	NYHA class III-IV, LVEF% ≤0.35	Carvedilol (25 mg BID) versus placebo	Treatment: 33; Placebo: 16
Effect of metoprolol CR/XL in chronic heart failure: Metoprolol CR/XL Randomised Intervention Trial in Congestive Heart Failure (MERIT-HF)	MERIT-HF	MERIT study group	1999	Lancet. 1999;353 (9169): 2001–7	Age 40–80 y, NYHA class II–IV, LVEF ≤ 0.40, HR > 68 beats/min, SBP > 100 mmHg	Metoprolol CR/XL (target: 200 mg/day) versus placebo	Treatment: 1990; Placebo: 2001
Carvedilol produces dose-related improvements in left ventricular function and survival in subjects with chronic heart failure	MOCHA	Bristow	1996	Circulation. 1996;94(11): 2807–16.	Age 18–85 y, NYHA class II–III, LVEF% ≤0.35, HR > 68 beats/min, SBP > 85 mmHg	Carvedliol (6.25, 12.5, or 25 mg BID) versus placebo	Treatment: 83/89/89; Placebo: 84
Carvedilol improves left ventricular function and symptoms in chronic heart failure: a double-blind randomized study	–	Olsen	1995	J Am Coll Cardiol. 1995;25(6): 1225–31.	Age 18–80 y, NYHA class II–III, LVEF% ≤0.35	Carvedilol (target 25–50 mg/day) versus placebo	Treatment: 36; Placebo: 24
Double-blind, placebo-controlled study of the effects of carvedilol in patients with moderate to severe heart failure	PRECISE	Packer	1996	Circulation. 1996;94(11): 2793–9.	NYHA class II–IV, LVEF ≤ 0.35, HR > 68 beats/min, SBP > 85 mmHg	Carvedilol (25–50 mg/day) versus placebo	Treatment: 133; Placebo: 145
Randomized trial to determine the effect of nebivolol on mortality and cardiovascular hospital admission in elderly patients with heart failure (SENIORS)	SENIORS	Flather	2005	Eur Heart J. 2005;26(3): 215–25.	Age ≥ 70 y, NYHA class I–IV, LVEF% ≤0.35, HR > 60 beats/min, SBP > 90 mmHg	Nebivolol (10 mg/day) versus placebo	Treatment: 1067; Placebo: 1061
Ivabradine and outcomes in chronic heart failure (SHIFT): a randomised placebo-controlled study	SHIFT	Swedberg	2010	Lancet. 2010;376(9744): 875–85.	Age ≥ 18 y, NYHA class II–IV, LVEF% ≤0.35, HR > 70 beats/min	Ivabradine (target: 7.5 mg BID) versus placebo	Treatment: 3241; Placebo: 3264
Effect of β1 blockade with atenolol on progression of heart failure in patients pretreated with high dose enalapril	–	Sturm	2000	Eur J Heart Fail. 2000;2(4): 407–12.	NYHA class II–III, LVEF% ≤0.25	Atenolol (target: 50–100 mg/day) versus placebo	Treatment: 51; Placebo: 49
The effect of carvedilol on morbidity and mortality in patients with chronic heart failure	US-CHF	Packer	1996	N Engl J Med. 1996; 334(21): 1349–55.	LVEF% ≤0.35, HR > 68 beats/min, SBP < 160 mmHg	Carvedilol (target: 6.25 mg, 12.5 mg, or 25 mg BID) versus placebo	Treatment: 696; Placebo: 398

BID: twice a day; HR: heart rate; LVEF: left ventricular ejection fraction; NYHA: New York Heart Association; SBP: systolic blood pressure.

All studies reported adequate randomization, although most studies had an unclear risk of bias due to the absence of details that were related primarily to random sequence generation, allocation concealment, and blinding of participants. Moreover, a high risk of bias was reported in 12 out of 20 studies due to industry sponsorship [18], [19], [20], [23], [24], [26], [27], [28], [30], [31], [32], [35]. Selective reporting (n = 5) [12], [24], [26], [29], [33] and incomplete outcome data (n = 5) [12], [21], [25], [32], [34] also contributed to a high risk of bias in other studies.

### 3.2 Bayesian random effect *meta*-analysis

In this analysis, we included 23,564 patients from 20 studies, and based on the available data, the HF characteristics analyzed included AF, ischemia (grouped as patients with a prior MI or diagnosed with either ischemia or coronary artery disease), non-ischemia, and a NYHA classification of II, III, and IV. We also analyzed a HR reduction ≥ 10 bpm as well as the comorbid presence of T2DM and hypertension.

**Fig. 1:**
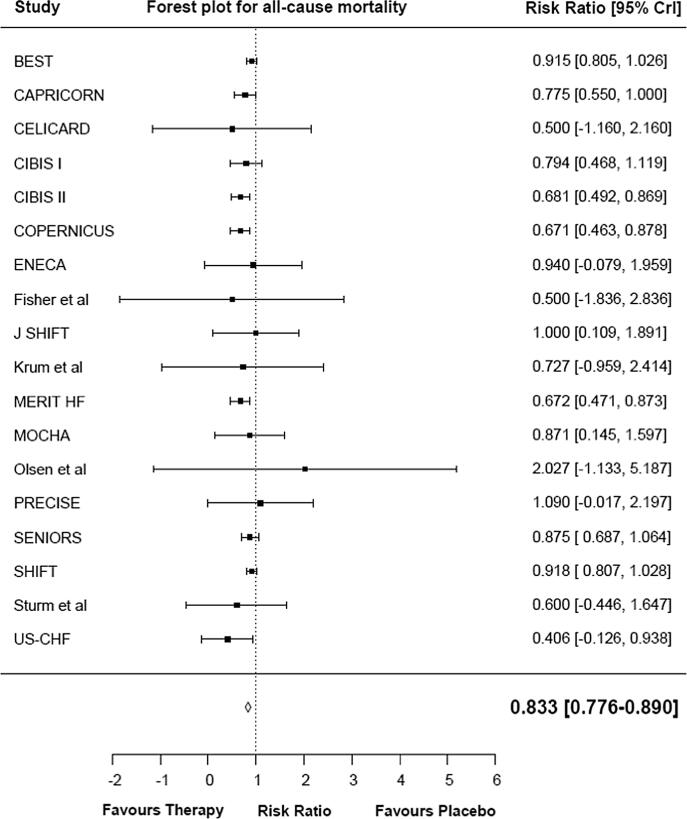

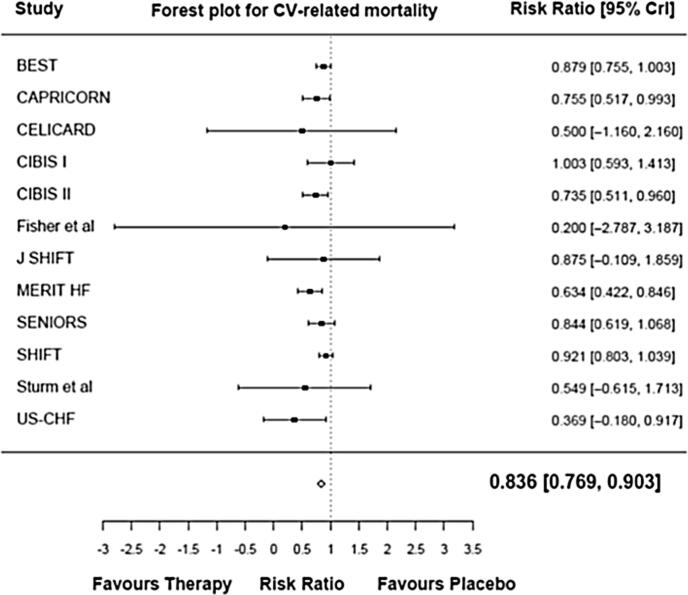

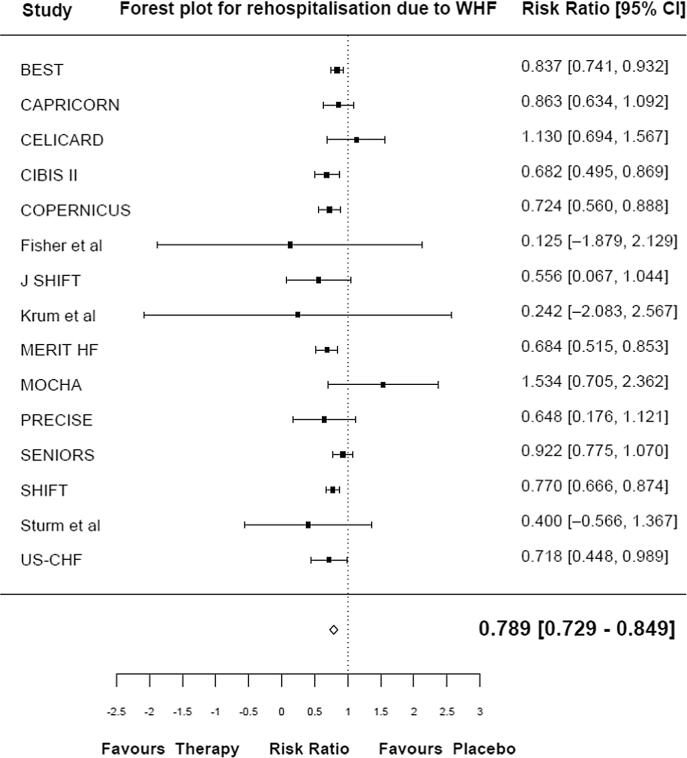
Forest plot for all-cause mortality (a), CV-related mortality (b), and rehospitalization due to WHF (c). CrI: credible interval; CV: cardiovascular; WHF: worsening heart failure.

The data were pooled using a Bayesian random effect *meta*-analysis (empirical Bayes) to estimate the overall effect on all-cause mortality, CV-related mortality, and rehospitalization due to WHF. The empirical Bayes model showed that HR reduction therapy was associated with a 16.7 % reduction in the risk of all-cause mortality, relative to placebo (RR 0.833 [95 % CrI 0.776, 0.890]) (Fig. 1**a**). HR reduction therapy was associated with a 16.4 % reduction in the risk of CV mortality in the pooled analysis, relative to placebo (RR 0.836; [95 % CrI 0.769, 0.903]) (Fig. 1**b**). HR reduction therapy was associated with a 21.1 % reduction in the risk of rehospitalization due to WHF in the pooled analysis, relative to placebo (RR 0.789 [95 % CrI 0.729, 0.849]) (Fig. 1**c**).

A leave-one-out analysis was performed to cross-validate the results from the Bayesian random effects model and to further assess the sensitivity and the risk of bias in individual studies (**Tables A.2–4**). The point estimate RRs remained consistent in terms of both magnitude and significance (all *p* < 0.01), regardless of which study was omitted, and no individual study significantly affected the overall result.

### 3.3 Empirical Bayes random effect *meta*-regression

**Fig. 2:**
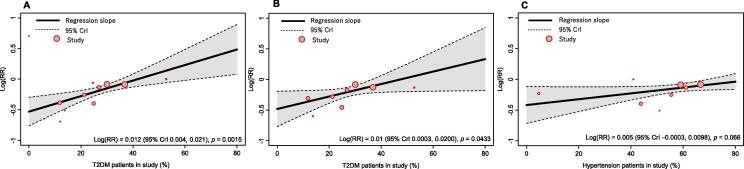
Meta-regression plots for T2DM and all-cause mortality (a), T2DM and CV-related mortality (b), and hypertension and all-cause mortality (c). CrI: credible interval; RR: risk ratio; T2DM: type 2 diabetes mellitus.

An empirical Bayes random effect *meta*-regression showed that T2DM was a statistically significant predictive factor for increasing the risk of all-cause mortality (log[RR] 0.012 [95 % CrI 0.004, 0.021], *p* = 0.0015) and CV-related mortality (log[RR] 0.01 [95 % CrI 0.0003, 0.0200], *p* = 0.043) in patients treated with HR-reducing therapy (Fig. 2**a and 2b; Table A.5**). When looking for other factors of interest (*p* < 0.1), it was observed that the presence of hypertension showed an increased risk of all-cause mortality in patients treated with HR-reducing therapy (log[RR] 0.005 [95 % CrI − 0.0003, 0.0098], *p* = 0.066) (Fig. 2**c; Table A.5**).

### 3.4 Subgroup analysis stratified by a HR reduction of < 10 bpm

**Fig. 3:**
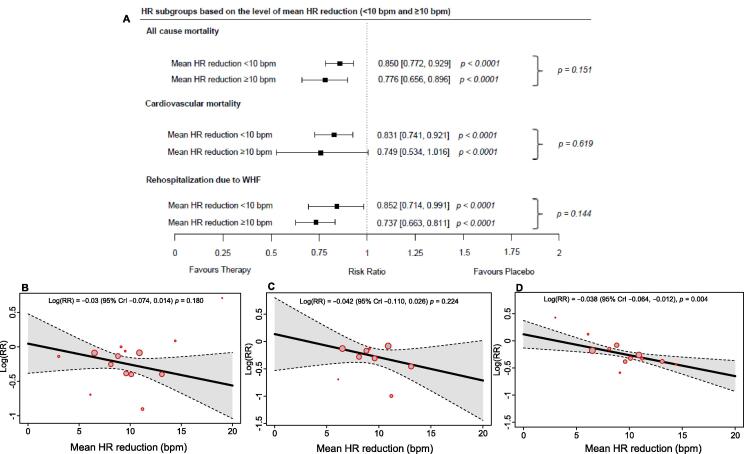
Forest plot for HR subgroups (a) and *meta*-regression plots for HR reduction and all-cause mortality (b), CV-related mortality (c), and rehospitalization due to WHF (d). bpm: beats per minute; CrI: credible interval; CV: cardiovascular; HR: heart rate; RR: risk ratio; WHF: worsening heart failure. An Omnibus test was used for comparing outcome risk for HR reduction < 10 bpm versus HR reduction ≥ 10 bpm.

When estimating the overall effect of therapy when stratified by a HR reduction of < 10 bpm or ≥ 10 bpm, we show a significant 15.0 % and 22.4 % reduction in the risk of all-cause mortality in the therapy group, relative to placebo (RR 0.850 [95 % CrI 0.772, 0.929], *p* < 0.0001 and RR 0.776 [95 % CrI 0.656, 0.896], *p* < 0.0001) (Fig. 3**a**). Furthermore, we also observed that there were significant reductions (16.9 % and 25.1 %, respectively) in the risk of CV-related mortality (RR 0.831 [95 % CrI 0.741, 0.921], *p* < 0.0001 and RR 0.749 [95 % CrI 0.534, 1.016], *p* < 0.0001), and significant reductions (14.8 % and 26.3 %) in the risk of rehospitalization due to WHF (RR 0.852 [95 % CrI 0.714, 0.991], *p* < 0.0001 and RR 0.737 [95 % CrI 0.663, 0.811], *p* < 0.0001). *Meta*-regression analysis of HR reduction showed non-significant trends in the slope profile for all-cause mortality and CV-related mortality risk per bpm (*p* = 0.180 and *p* = 0.224, respectively) (Fig. 3**b, c**). However, for rehospitalization due to WHF we observed a significant reduction in risk per bpm reduced (*p* = 0.004) (Fig. 3**d**). There were insufficient studies to perform a *meta*-regression analysis when stratifying by a HR reduction threshold of 10 bpm.

### 3.5 Publication bias

Funnel plots and the Egger’s test were used to assess publication bias (Fig. A.2**a–c**). All studies lie within the geometric threshold for plot asymmetry, which indicates that there was no plot asymmetry or publication bias. Egger’s test *p* values were all > 0.05, which also indicates that there was no publication bias in any of the *meta*-analyses.

## 4 Discussion

We conducted the present *meta*-analysis using a Bayesian approach to evaluate patient and disease-related factors that interact with HR-reducing therapy on the clinical outcomes in patients with HFrEF. In this *meta*-analysis, we showed that HR-reducing therapy was associated with significant reductions in the risk of all-cause mortality, CV-related mortality, and rehospitalization due to WHF. Furthermore, we evaluated nine potential predictors (atrial fibrillation, T2DM, hypertension, LVEF, ischemia, NYHA class [I-III], and HR reduction) and showed that the presence of T2DM significantly modifies the effect of HR-reducing therapy on the risk of all-cause mortality and CV-related mortality.

In this study, the presence of T2DM was associated with an increase in the relative risk of all-cause mortality and CV-related mortality. Patients with T2DM not only have a higher risk of developing HF, but also their CV outcomes, hospitalization rates, and prognoses are substantially worse than those without T2DM [37], [38]. Although previous *meta*-analyses support the benefit of HR reduction in HF patients with T2DM, it has been reported that the magnitude of benefit may be somewhat reduced in chronic HF patients with T2DM [39]. Interestingly, the study by Haas et al used data from CIBIS-II, BEST, ANZ, US-CHF, COPERNICUS, and MERIT-HF, which is a small subset of the studies included in the present analysis [39]. There is a complex and interrelated pathophysiology of HF in T2DM due to the dysregulation of several mechanisms; for instance, research shows that HF development in T2DM patients is strongly influenced by hyperglycemia [40] and obesity [41]. Furthermore, T2DM also directly impacts the myocardium leading to progressive structural and functional changes (i.e., diabetic cardiomyopathy) [42]. Overall, the cardiac changes observed in T2DM include increased interstitial fibrosis, increased LV wall thickness, functional myocardial impairments, and chronological impairment due to cardiovascular autonomic neuropathy [43], which together are likely to reduce the benefits achieved by HR reduction.

The *meta*-regression plot for T2DM’s influence on mortality suggests that our observations are well represented by the linear trend in the data. When comparing the all-cause mortality *meta*-regression plots for T2DM and hypertension, we can see that for T2DM each study included is near the straight line, whereas for hypertension there is a greater degree of heterogeneity, suggesting this slope profile may not be as robust. Furthermore, when comparing the angle of these slope profiles, we can determine that T2DM has a stronger moderating effect on the relative risk for all-cause mortality compared with hypertension. Although this *meta*-regression plot showed that the percentage of patients was generally greater and there was a non-significant trend, it is not clear whether the log(RR) for hypertension is indicative of an increasing effect on intervention efficacy. Certainly, high blood pressure is an important risk factor for CV disease and mortality, and a reduction in cardiac burden, as elicited by HR reduction therapy, should be more beneficial in this patient subgroup [44]. A previous *meta*-analysis did identify that a beta-blocker-associated reduction in HR increased the risk of mortality in patients with hypertension compared with patients with HF [45]. However, the *meta*-analysis by Bangalore et al primarily included studies that administered atenolol and as such this observation may not be a class effect but instead a specific effect of the soluble beta blocker, atenolol. Overall, the relationship between HR reduction and comorbid hypertension is complicated in this patient population and although there was a trend, our results do not support any significant interaction.

Finally, we also showed that HR reduction ≥ 10 bpm conferred a non-significant greater reduction in risk for all-cause mortality, CV-related mortality, and rehospitalization due to WHF in comparison with a HR reduction of < 10 bpm. The magnitude of HR reduction has previously been shown to be significantly associated with survival benefit, whereas the dose of beta-blockers was not associated with survival benefit [10]. Therefore, our results support previous observations that a greater reduction in HR is associated with a greater reduction in mortality. When performing a *meta*-regression, the regression slope profile was indicative of a reduction in risk for each outcome, but this was only significant for rehospitalization due to WHF. Here, our data show that as mean HR is progressively reduced there is a significant association with greater reductions in the risk of rehospitalization due to WHF.

### 4.1 Study limitations

Although our *meta*-analysis included all available randomized controlled trials, there were insufficient data to conduct any subgroup sensitivity analyses. Second, this was a publication-based *meta*-regression and as such our observations might be influenced by ecological bias [46]. Third, because of the limited number of studies, this *meta*-regression was not a multivariable regression and thus the association of each possible predictor might be confounded by other prognostic factors. Finally, although we assessed the risk of bias as acceptable, there were a substantial number of biases graded as unclear. However, when exploring the potential risk of bias across the studies, publication bias was excluded using Egger’s test.

## 5 Conclusions

We observed that the presence of comorbid T2DM in HFrEF patients significantly reduces the benefit of HR reduction, while for comorbid hypertension, there was a non-significant trend for a reduced benefit from HR reduction. As such, the patient populations that would benefit the most from HR reducing therapy are HFrEF patients without comorbid diabetes or hypertension, although only the former was significant. The absence of more factors exhibiting significance is potentially indicative of HR reduction being the most important treatment modality in HFrEF patients. However, we are also aware that our study did not include individual patient data and as such more research is required to further elucidate the predictive nature of the factors evaluated in our study to affect clinical outcomes in patients with HFrEF.

## Declaration of Competing Interest

The authors declare that they have no known competing financial interests or personal relationships that could have appeared to influence the work reported in this paper.
