# Supplementary material for: Identification of predictive factors interacting with heart rate reduction for potential beneficial clinical outcomes in chronic heart failure: A systematic literature review and meta-analysis
Source: Int J Cardiol Heart Vasc. 2022 Oct 29;43:101141. doi: 10.1016/j.ijcha.2022.101141 (PMC9634015; doi:10.1016/j.ijcha.2022.101141)
Supplement: Supplementary data 1 [file mmc1.docx]

# Appendix A: Supplementary data

**Fig. A.1** The PRISMA flow diagram.

**Fig. A.2** Funnel plot for all-cause mortality (a), CV-related mortality (b), and rehospitalization due to WHF (c).

CV: cardiovascular; WHF: worsening heart failure.

**Text A.1** PRISMA checklist.

**Table A.1** Post-hoc analyses.

| **Title** | **Trial Name** | **First Author** | **Year** | **Citation** |
| --- | --- | --- | --- | --- |
| Heart rate at baseline influences the effect of ivabradine on cardiovascular outcomes in chronic heart failure: analysis from the SHIFT study | SHIFT | Böhm | 2013 | Clin Res Cardiol. 2013;102(1): 11-22. |
| Heart rate as a risk factor in chronic heart failure (SHIFT): the association between heart rate and outcomes in a randomized placebo-controlled trial | SHIFT | Böhm | 2010 | Lancet. 2010;376(9744): 886-94. |
| Influence of cardiovascular and noncardiovascular co-morbidities on outcomes and treatment effect of heart rate reduction with ivabradine in stable heart failure (from the SHIFT Trial) | SHIFT | Böhm | 2015 | Am J Cardiol. 2015;116(12): 1890-7. |
| Heart rate reduction with ivabradine and health related quality of life in patients with chronic heart failure: results from the SHIFT study | SHIFT | Ekman | 2011 | Eur Heart J. 2011;32(19): 2395-404. |
| Top ten risk factors for morbidity and mortality in patients with chronic systolic heart failure and elevated heart rate: The SHIFT risk model | SHIFT | Ford | 2015 | Int J Cardiol. 2015;184: 163-9. |
| Analyzing health-related quality of life data to estimate parameters for cost-effectiveness models: an example using longitudinal EQ-5D data from the SHIFT randomized controlled trial | SHIFT | Griffiths | 2017 | Adv Ther. 2017;34(3): 753-764. |
| Effects on outcomes of heart rate reduction by ivabradine in patients with congestive heart failure: is there an influence of beta-blocker dose?: findings from the SHIFT (Systolic Heart failure treatment with the I(f) inhibitor ivabradine trial) study | SHIFT | Swedberg | 2012 | J Am Coll Cardiol. 2012;59(22): 1938-45. |
| The effect of heart rate reduction with ivabradine on renal function in patients with chronic heart failure: an analysis from SHIFT | SHIFT | Voors | 2014 | Eur J Heart Fail. 2014;16(4): 426-34. |
| Effects of Carvedilol on Left Ventricular Remodeling After Acute Myocardial Infarction | CAPRICORN | Doughty | 2004 | Circulation. 2004;109(2): 201-6 |
| Landmark study: the Carvedilol Post-Infarct Survival Control in Left Ventricular Dysfunction Study (CAPRICORN) | CAPRICORN | Colucci | 2004 | Am J Cardiol. 2004;93(9A): 13B-6B. |
| Antiarrhythmic effect of carvediolol after acute myocardial infarction: results of the Carvedilol Post-Infarct Survival Control in Left Ventricular Dysfunction (CAPRICORN) trial | CAPRICORN | McMurray | 2005 | J Am Coll Cardiol. 2005;45(4): 525-30. |
| Effects of controlled-release metoprolol on total mortality, hospitalizations, and well-being in patients with heart failure: the metoprolol CR/XL randomized intervention trial in congestive heart failure (MERIT-HF). MERIT-HF Study Group | MERIT-HF | Hjalmarson | 2000 | JAMA. 2000;283(10): 1295-302. |
| Dose of metoprolol CR/XL and clinical outcomes in patients with heart failure: Analysis of the experience in metoprolol CR/XL randomized intervention trial in chronic heart failure (MERIT-HF) | MERIT-HF | Wikstrand | 2002 | J Am Coll Cardiol. 2002;40(3): 491-8. |
| Metoprolol CR/XL in black patients with heart failure (from the metoprolol CR/XL randomized intervention trial in chronic heart failure) | MERIT | Goldstein | 2003 | Am J Cardiol. 2003;92(4): 478-80. |
| Tolerability of β-Blocker Initiation and titration in the metoprolol CR/XL randomized intervention trial in congestive heart failure (MERIT-HF) | MERIT | Gottlieb | 2002 | Circulation.2002;105(10): 1182-8. |
| Metoprolol CR/XL in female patients with heart failure | MERIT | Ghali | 2002 | Circulation. 2002;105(13): 1585-91 |
| Effect of carvedilol on the morbidity of patients with severe chronic heart failure: results of the carvedilol prospective randomized cumulative survival (COPERNICUS) study | COPERNICUS | Packer | 2002 | Circulation. 2002;106(17): 2194-9 |
| Carvedilol prospective randomized cumulative survival (COPERNICUS) trial: carvedilol in severe heart failure | COPERNICUS | Fowler | 2004 | Am J Cardiol 2004;93(suppl): 35B–39B |
| Effect of carvedilol on survival in severe chronic heart failure | COPERNICUS | Packer | 2001 | N Engl J Med. 2001 31;344(22): 1651-8 |
| Effects of initiating carvedilol in patients with severe chronic heart failure: results from the COPERNICUS study | COPERNICUS | Krum | 2003 | JAMA. 2003;289(6):712-718. |
| Influence of pretreatment systolic blood pressure on the effect of carvedilol in patients with severe chronic heart failure: the carvedilol prospective randomized cumulative survival (COPERNICUS) study | COPERNICUS | Rouleau | 2004 | J Am Coll Cardiol. 2004;43(8): 1423-9. |
| Re-examination of the BEST trial using composite outcomes, including emergency department visits | BEST | Shen | 2017 | JACC Heart Fail. 2017;5(8): 591-599. |
| Effect of bucindolol on heart failure outcomes and heart rate response in patients with reduced ejection fraction heart failure and atrial fibrillation | BEST | Kao | 2013 | Eur J Heart Fail. 2013;15(3): 324-33. |
| Bucindolol, systolic blood pressure, and outcomes in systolic heart failure: a prespecified post hoc analysis of BEST | BEST | White | 2012 | Can J Cardiol. 2012;28(3): 354–359. |
| Results from post-hoc analyses of the CIBIS II trial: effect of bisoprolol in high-risk patient groups with chronic heart failure | CIBIS II | Erdmann | 2001 | Eur Heart J. 2001;3(4): 469-79. |
| Bisoprolol dose–response relationship in patients with congestive heart failure: a subgroup analysis in the cardiac insufficiency bisoprolol study (CIBIS II) | CIBIS II | Simon | 2003 | Eur Heart J. 2003;24(6): 552-9 |
| Improved survival with bisoprolol in patients with heart failure and renal impairment: an analysis of the cardiac insufficiency bisoprolol study II (CIBIS‐II) trial | CIBIS II | Castagno | 2010 | Eur J Heart Fail. 2010;12(6): 607-16. |
| Carvedilol Inhibits Clinical Progression in Patients with Mild Symptoms of Heart Failure | US-CHF | Colucci | 1996 | Circulation. 1996; 94:2800–2806 |
| Safety and efficacy of carvedilol in severe heart failure. The U.S. Carvedilol Heart Failure Study Group | US-CHF | Cohn | 1997 | J Card Fail. 1997; 3(3): 173-9. |
| Influence of carvedilol on hospitalizations in heart failure: incidence, resource utilization and costs. US Carvedilol Heart Failure Study Group. | US-CHF | Fowler | 2001 | J Am Coll Cardiol. 2001;37(6): 1692-9. |
| Efficacy and safety of nebivolol in elderly heart failure patients with impaired renal function: insights from the SENIORS trial | SENIORS | Cohen-Solal | 2009 | Eur J Heart Fail. 2009;11(9): 872–880. |
| Effects of nebivolol in elderly heart failure patients with or without systolic left ventricular dysfunction: results of the SENIORS echocardiographic substudy | SENIORS | Ghio | 2006 | Eur Heart J. 2006;27(5): 562-8 |
| β-blockade with nebivolol for prevention of acute ischemic events in elderly patients with heart failure | SENIORS | Ambrosio | 2011 | Heart. 2011;97(3):209-14. |

**Table A.2** Leave-one-out analysis of therapy effect versus all-cause mortality.

| Study Omitted | RR | z | *p* value | Lower  95% CrI | Upper  95% CrI | *I*^2^ (%) |
| --- | --- | --- | --- | --- | --- | --- |
| BEST | 0.80 | 23.65 | <0.01 | 0.74 | 0.87 | <0.01 |
| CAPRICORN | 0.84 | 27.83 | <0.01 | 0.78 | 0.90 | <0.01 |
| CELICARD | 0.83 | 28.62 | <0.01 | 0.78 | 0.89 | <0.01 |
| CIBIS I | 0.83 | 28.20 | <0.01 | 0.78 | 0.89 | 0.04 |
| CIBIS II | 0.85 | 27.79 | <0.01 | 0.79 | 0.91 | <0.01 |
| Fisher et al | 0.83 | 28.63 | <0.01 | 0.78 | 0.89 | <0.01 |
| J SHIFT | 0.83 | 28.55 | <0.01 | 0.78 | 0.89 | <0.01 |
| MERIT HF | 0.85 | 27.92 | <0.01 | 0.79 | 0.91 | <0.01 |
| SENIORS | 0.83 | 27.15 | <0.01 | 0.77 | 0.89 | <0.01 |
| SHIFT | 0.80 | 23.63 | <0.01 | 0.74 | 0.87 | <0.01 |
| Sturm et al | 0.83 | 28.61 | <0.01 | 0.78 | 0.89 | <0.01 |
| US-CHF | 0.84 | 28.63 | <0.01 | 0.78 | 0.90 | <0.01 |

CrI: credible interval; RR: risk ratio; *I*^2^: between study heterogeneity.

**Table A.3** Leave-one-out analysis of therapy effect versus cardiovascular-related mortality.

| Study Omitted | RR | z | *p* value | Lower  95% CrI | Upper  95% CrI | *I*^2^ (%) |
| --- | --- | --- | --- | --- | --- | --- |
| BEST | 0.82 | 19.65 | <0.01 | 0.73 | 0.90 | 2.21 |
| CAPRICORN | 0.84 | 22.30 | <0.01 | 0.77 | 0.91 | 4.29 |
| CELICARD | 0.83 | 22.53 | <0.01 | 0.76 | 0.90 | 6.67 |
| CIBIS I | 0.83 | 23.19 | <0.01 | 0.76 | 0.90 | 2.92 |
| CIBIS II | 0.84 | 23.14 | <0.01 | 0.77 | 0.92 | 1.86 |
| Fisher et al | 0.83 | 22.58 | <0.01 | 0.76 | 0.90 | 6.54 |
| J SHIFT | 0.83 | 22.16 | <0.01 | 0.76 | 0.90 | 7.69 |
| MERIT HF | 0.86 | 24.09 | <0.01 | 0.79 | 0.93 | <0.01 |
| SENIORS | 0.83 | 21.11 | <0.01 | 0.75 | 0.90 | 6.77 |
| SHIFT | 0.80 | 19.53 | <0.01 | 0.72 | 0.88 | <0.01 |
| Sturm et al | 0.83 | 22.68 | <0.01 | 0.76 | 0.90 | 6.13 |
| US-CHF | 0.84 | 24.74 | <0.01 | 0.78 | 0.91 | <0.01 |

CrI: credible interval; RR: risk ratio; *I*^2^: between study heterogeneity.

**Table A.4** Leave-one-out analysis of therapy effect versus rehospitalization due to worsening heart failure.

| Study Omitted | RR | z | *p* value | Lower  95% CrI | Upper  95% CrI | *I*^2^ (%) |
| --- | --- | --- | --- | --- | --- | --- |
| BEST | 0.78 | 21.69 | <0.01 | 0.71 | 0.85 | 14.42 |
| CAPRICORN | 0.78 | 23.33 | <0.01 | 0.72 | 0.85 | 20.06 |
| CELICARD | 0.79 | 28.48 | <0.01 | 0.73 | 0.84 | 5.44 |
| CIBIS II | 0.80 | 25.28 | <0.01 | 0.74 | 0.86 | 13.18 |
| COPERNICUS | 0.80 | 23.40 | <0.01 | 0.73 | 0.86 | 18.17 |
| Fisher et al | 0.79 | 24.40 | <0.01 | 0.73 | 0.85 | 20.66 |
| J SHIFT | 0.79 | 25.31 | <0.01 | 0.73 | 0.85 | 16.70 |
| Krum et al | 0.79 | 23.94 | <0.01 | 0.72 | 0.85 | 22.49 |
| MERIT HF | 0.80 | 25.64 | <0.01 | 0.74 | 0.86 | 11.26 |
| MOCHA | 0.79 | 30.69 | <0.01 | 0.74 | 0.84 | 0.35 |
| PRECISE | 0.79 | 24.05 | <0.01 | 0.73 | 0.85 | 21.22 |
| SENIORS | 0.78 | 28.53 | <0.01 | 0.72 | 0.83 | <0.01 |
| SHIFT | 0.79 | 21.41 | <0.01 | 0.72 | 0.86 | 18.90 |
| Sturm et al | 0.79 | 24.83 | <0.01 | 0.73 | 0.85 | 18.93 |
| US-CHF | 0.79 | 23.48 | <0.01 | 0.73 | 0.86 | 21.51 |

CrI: credible interval; RR: risk ratio; *I*^2^: between study heterogeneity.

Table A.5 Bayesian random effect meta-regression for all-cause mortality, CV-related mortality, and rehospitalization due to WHF.

|  | **log(RR)** | **95% CrI**  **(Lower)** | **95% CrI**  **(Upper)** | **PP** | ***p* value** | **Patients (Studies)** |
| --- | --- | --- | --- | --- | --- | --- |
| **All-cause mortality** |  |  |  |  |  |  |
| **Covariates** |  |  |  |  |  |  |
| AF | -0.0007 | -0.009 | 0.008 | 0.127 | 0.873 | 21,243 (n=11) |
| T2DM | 0.012 | 0.004 | 0.021 | 0.998 | 0.0015 | 20,662 (n=11) |
| HR reduction ≥10bpm | -0.18 | -0.410 | 0.060 | 0.860 | 0.140 | 25,111 (n=15) |
| Hypertension | 0.005 | -0.0003 | 0.0098 | 0.934 | 0.066 | 18,546 (n=9) |
| LVEF (per %) | 0.01 | -0.005 | 0.04 | 0.850 | 0.150 | 25,310 (n=18) |
| Ischemia Composite | -0.001 | -0.005 | 0.003 | 0.340 | 0.660 | 25,310 (n=18) |
| NYHA II | 0.0005 | -0.010 | 0.010 | 0.050 | 0.950 | 15,066 (n=13) |
| NYHA III | 0.001 | -0.004 | 0.007 | 0.290 | 0.710 | 22,994 (n=14) |
| NYHA IV | -0.001 | -0.006 | 0.003 | 0.446 | 0.554 | 22,994 (n=14) |
|  |  |  |  |  |  |  |
| **CV-related mortality** | | |  |  |  |  |
| **Covariates** |  |  |  |  |  |  |
| AF | -0.002 | -0.013 | 0.009 | 0.235 | 0.765 | 20,342 (n=9) |
| T2DM | 0.01 | 0.0003 | 0.020 | 0.957 | 0.043 | 20,342 (n=9) |
| HR reduction ≥10bpm | -0.149 | -0.531 | 0.232 | 0.556 | 0.444 | 21,410 (n=9) |
| Hypertension | -0.0012 | -0.008 | 0.004 | 0.450 | 0.550 | 17,645 (n=8) |
| LVEF (per %) | 0.024 | -0.011 | 0.058 | 0.820 | 0.180 | 21,560 (n=11) |
| Ischemia Composite | -0.0009 | -0.006 | 0.004 | 0.238 | 0.762 | 21,560 (n=11) |
| NYHA II | 0.002 | -0.018 | 0.023 | 0.183 | 0.817 | 14,246 (n=8) |
| NYHA III | 0.004 | -0.002 | 0.012 | 0.792 | 0.208 | 19,477 (n=8) |
| NYHA IV | 0.003 | -0.040 | 0.047 | 0.118 | 0.882 | 19,477 (n=8) |
| **Rehospitalization due to WHF** | | |  |  |  |  |
| **Covariates** |  |  |  |  |  |  |
| AF | -0.0004 | -0.022 | 0.022 | 0.03 | 0.970 | 20,342 (n=9) |
| T2DM | 0.002 | -0.015 | 0.019 | 0.153 | 0.847 | 20,342 (n=10) |
| HR reduction ≥10bpm | -0.198 | -0.468 | 0.072 | 0.849 | 0.151 | 24,150 (n=12) |
| Hypertension | 0.015 | -0.002 | 0.022 | 0.926 | 0.119 | 17,645 (n=7) |
| LVEF (per %) | 0.018 | -0.015 | 0.052 | 0.721 | 0.279 | 24,349 (n=15) |
| Ischemia Composite | 0.0009 | -0.010 | 0.012 | 0.121 | 0.879 | 24,349 (n=15) |
| NYHA II | -0.005 | -0.025 | 0.014 | 0.41 | 0.590 | 14,746 (n=11) |
| NYHA III | 0.003 | -0.006 | 0.013 | 0.488 | 0.512 | 22,093 (n=12) |
| NYHA IV | 0.0004 | -0.004 | 0.005 | 0.138 | 0.862 | 22,093 (n=12) |

AF: atrial fibrillation; CrI: credible interval; HR: heart rate; LVEF: left ventricular ejection fraction; NYHA: New York Heart Association; PP: posterior probability; RR: risk ratio; T2DM: type 2 diabetes mellitus; WHF: worsening due to heart failure.
