# Supplementary material for: Identification of predictive factors interacting with heart rate reduction for potential beneficial clinical outcomes in chronic heart failure: A systematic literature review and meta-analysis
Source: Int J Cardiol Heart Vasc. 2022 Oct 29;43:101141. doi: 10.1016/j.ijcha.2022.101141 (PMC9634015; doi:10.1016/j.ijcha.2022.101141)
Supplement: Supplementary data 3 [file mmc3.pptx]

## Slide 1
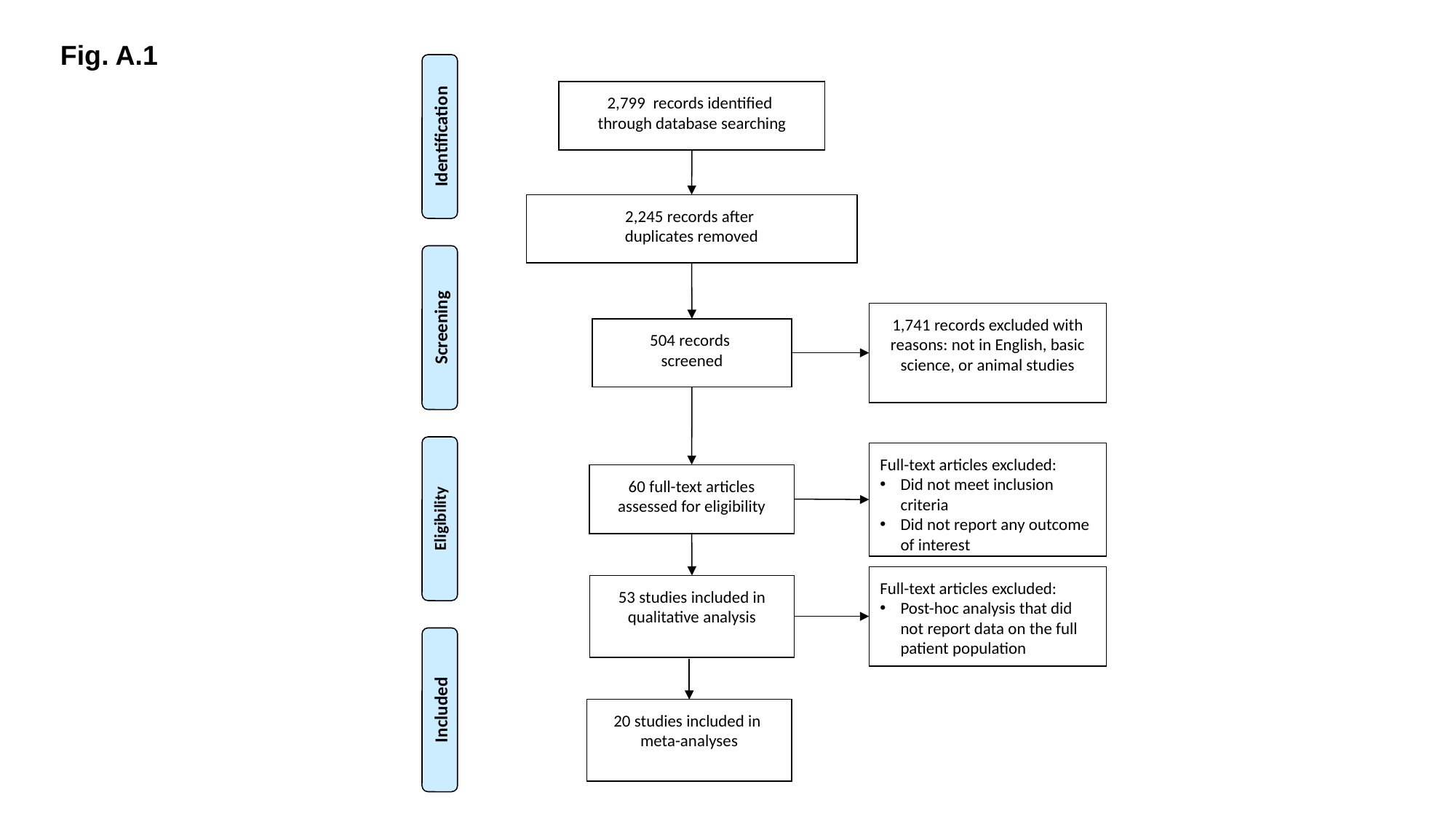

Fig. A.1
2,799 records identified
through database searching
Identification
2,245 records after
duplicates removed
1,741 records excluded with reasons: not in English, basic science, or animal studies
Screening
504 records
screened
Full-text articles excluded:
Did not meet inclusion criteria
Did not report any outcome of interest
60 full-text articles assessed for eligibility
Eligibility
Full-text articles excluded:
Post-hoc analysis that did not report data on the full patient population
53 studies included in qualitative analysis
Included
20 studies included in
meta-analyses
