# Supplementary figures and images for: Identification of predictive factors interacting with heart rate reduction for potential beneficial clinical outcomes in chronic heart failure: A systematic literature review and meta-analysis
Source: Int J Cardiol Heart Vasc. 2022 Oct 29;43:101141. doi: 10.1016/j.ijcha.2022.101141 (PMC9634015; doi:10.1016/j.ijcha.2022.101141)

## Slide 1
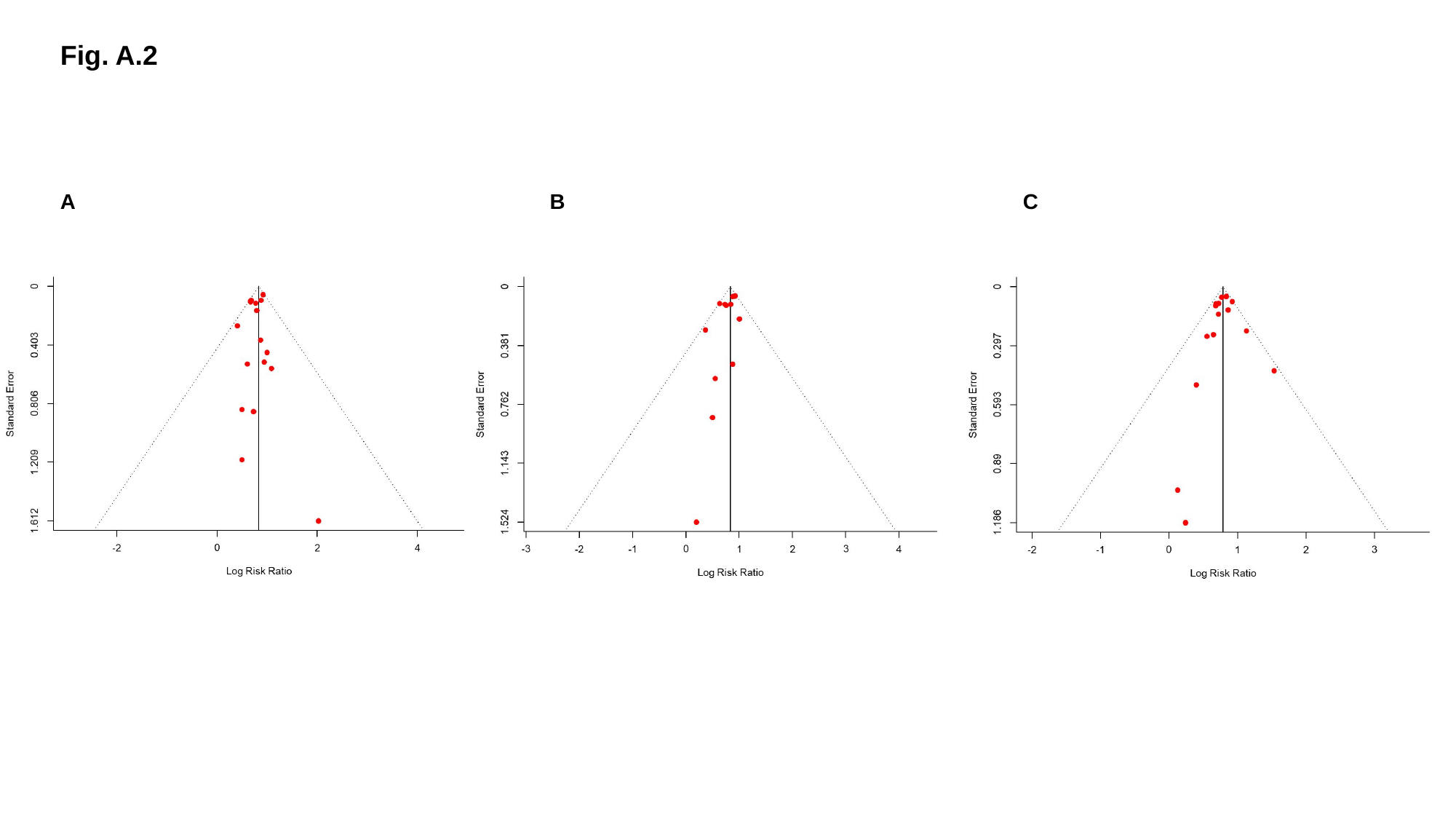

Fig. A.2
B
C
A

Supplement: Supplementary data 4 [file mmc4.pptx]
